# Seropositivity and Higher Immunoglobulin G Antibody Levels Against Cytomegalovirus Are Associated With Mortality in the Population-Based European Prospective Investigation of Cancer–Norfolk Cohort

**DOI:** 10.1093/cid/cit083

**Published:** 2013-02-26

**Authors:** Effrossyni Gkrania-Klotsas, Claudia Langenberg, Stephen J. Sharp, Robert Luben, Kay-Tee Khaw, Nicholas J. Wareham

**Affiliations:** 1 Medical Research Council Epidemiology Unit, Institute of Metabolic Science, Cambridge; 2 Department of Public Health and Primary Care, Institute of Public Health, University of Cambridge, United Kingdom

**Keywords:** cytomegalovirus, cancer, mortality, cohort study, cardiovascular disease

## Abstract

***Background.*** The relationship between cytomegalovirus (CMV) infection and mortality among immunocompetent individuals is uncertain. We aimed to examine whether seropositivity for CMV and the level of CMV immunoglobulin G (IgG) antibody are associated with all-cause and cause-specific mortality.

***Methods.*** We used data from a random sample of 13 090 participants aged 40–79 years at recruitment in 1993–1997 to the European Prospective Investigation of Cancer–Norfolk population-based cohort study. We measured baseline IgG antibody levels against CMV. Death certificates were obtained for all participants who died before 31 March 2011. Codes for the underlying cause of death were used to investigate cause-specific mortality.

***Results.*** A total of 2514 deaths occurred during a mean follow-up of 14.3 years (SD, 3.3 years). Compared to seronegative participants (age- and sex-adjusted mortality rate, 12.4 [95% confidence interval {CI}, 11.3–13.2] per 1000 person-years at risk), rates increased across thirds of IgG antibody levels (score test of trend *P* < .0001). CMV seropositivity (prevalence 59%) was associated with increased all-cause mortality (age- and sex-adjusted hazard ratio [HR], 1.16 [95% CI, 1.07–1.26]), similarly in men and women (*P* for interaction = .52). The association persisted after additionally adjusting for measures of socioeconomic status and possible confounders. Cause-specific analyses suggested that increased mortality from cardiovascular disease (HR, 1.06 [95% CI, .91–1.24]), cancer (HR, 1.13 [95% CI, .98–1.31]), and other causes (HR, 1.23 [95% CI, 1.04–1.47) all appeared to contribute to the overall associations.

***Conclusions.*** Seropositivity and higher IgG antibody levels against CMV are associated with increased mortality and after adjustment for a range of potential confounders in the general population.

## Abstract (precis)

After adjustment for a range of possible confounders, cytomegalovirus seropositivity and cytomegalovirus immunoglobulin G antibody levels were associated with all-cause mortality in the EPIC-Norfolk population-based cohort study.

Cytomegalovirus (CMV) is a common herpesvirus that infects the majority of the population worldwide. After primary infection, usually an undifferentiated febrile illness, the virus remains latent, under the control of the immune system, and is asymptomatic for the lifetime of the host [1]. Evidence of past CMV infection is the presence of immunoglobulin G (IgG) antibodies for CMV in the peripheral blood (seropositivity). If CMV escapes immunological control and reactivates from latency [1], it can cause severe disease and excess mortality, as has been observed among severely unwell [2] and immunocompromised individuals [3, 4], CMV infection in transplantation has been associated with direct organ damage as well as indirect consequences, such as transplant-associated vasculopathy, which can manifest as coronary artery stenosis and graft rejection, and an increased risk of opportunistic infection [5].

However, experimental and epidemiological data also point to the direction of CMV as a potential factor in the development of cardiovascular disease among immunocompetent individuals [1]. Some studies have reported associations between CMV seropositivity or level of CMV IgG antibody and cardiovascular disease [6–8], cardiovascular or all-cause mortality [9–16], or even cancer [17]. CMV infection has been hypothesized to increase mortality risk through its association with cardiovascular disease and frequent silent CMV reactivations that are driving chronic inflammation [13]. An alternative explanation could be that the reported associations are subject to residual confounding, given the social patterning of CMV infection [18].

CMV is only one of many pathogens that have been in the past examined for their possible associations with cardiovascular diseases. The list of candidates includes other viruses like Coxsackieviruses as well as bacteria; however, CMV remains of major interest because of possible dose effects described above as well as biologic plausibility [19, 20], which raise the possibility that CMV might be causal for increased morbidity and mortality.

In the current study, we examine whether seropositivity for CMV and the level of CMV IgG antibody are associated with all-cause and cause-specific mortality among participants in a population-based cohort study and whether markers of inflammation and socioeconomic status confound any observed associations.

## METHODS

### Ethics Statement

All volunteers gave written informed consent, and the study was approved by the Norfolk Research Ethics Committee.

### Cohort Characteristics

We studied this association in the European Prospective Investigation of Cancer (EPIC)–Norfolk study, a well-described UK population-based cohort [21], which recruited 25 639 men and women between 1993 and 1997, who were aged 40–79 years at baseline. From the participants with available sera, we randomly selected 13 090 for measurement of CMV antibodies.

### Questionnaires and Biochemical and Hematological Analyses

Anthropometric measurements were taken, and a health and lifestyle questionnaire that included questions on housing, occupational social class, educational level, use of tobacco, and physical activity was completed at baseline. A validated 4-point ordered categorical physical activity index was used [21]. The participants were asked to report if they had ever been diagnosed with a “heart attack” (myocardial infarction) or stroke or diabetes. Individual data were linked with the East Anglia Cancer Registry database and participants were coded as having had a history of cancer if they had been diagnosed with any cancer except for nonmelanoma skin cancers at study entry.

Serum high-sensitivity C-reactive protein (CRP) was measured using a Dade Behring Dimension ARx automated system (Deerfield, Illinois) and ferritin with a 2-step time-resolved fluoroimmunoassay (Wallac Oy, Turku, Finland). All other biochemical and hematological indices were measured using standard assays [21].

### Mortality Ascertainment

All participants were flagged for death certification at the Office of National Statistics (United Kingdom) with vital status ascertained on the entire cohort, and mortality data for all EPIC-Norfolk participants were available up to 31 March 2011. Coding of death certificates was executed by trained nosologists according to the *International Classification of Diseases* (*ICD*), *Ninth* or *Tenth Revision*. All deaths with *ICD-9* codes were recoded into comparable *ICD-10* codes. Only underlying causes of death were used for these analyses. A “cardiovascular death” was defined as a death where the underlying cause of death belonged to *ICD-10* codes I10–I79 inclusive and a “cancer death” to codes C00–C97. The cardiovascular codes were selected to reflect postulated CMV pathogenetic mechanisms and included causes of death such as hypertensive heart disease, atherosclerosis including coronary and cerebrovascular disease, heart failure, aortic aneurysms, and thrombosis [22].

### CMV Measurements

CMV-specific IgG measurements were performed on sera from 13 090 participants, by the Cambridge University Hospitals Virology Laboratory, using an indirect chemiluminescence immunoassay (Liaison, Diasorin, Saluggia, Italy). The amount of isoluminol-antibody conjugate is measured by a photomultiplier as relative light units (RLUs). The machine, using an internal algorithm, converts RLUs to antibody levels. The coefficient of variation for the assay is <8%, specificity 99.65%, sensitivity 99.88%, and repeatability >98%. The assay compares favorably to other CMV IgG assays for confirmation of past CMV infections [23]. Samples were shipped to the laboratory in randomly ordered boxes, with no identifiers other than a barcode. A sample was defined as being negative, equivocal, or positive for CMV IgG antibody using the clinical antibody cutoffs of the assay (<0.4 IU/mL, 0.4–0.6 IU/mL, and >0.6 IU/mL, respectively). Ninety-one participants had equivocal test results and were excluded. Because of low variable missingness (<5% for all variables), no other exclusions were applied and models were performed on the maximum number possible. Results from 7113 women and 5886 men were available for analysis.

### Statistical Analyses

RLUs were standardized by calculating the difference from the mean and dividing by the standard deviation within the day of measurement, to account for any differences in assay performance from day to day. Standardized RLUs among participants with positive tests only were grouped into thirds of the distribution.

We summarized baseline characteristics within the cohort using means and standard deviations for continuous variables with an approximately symmetric distribution, medians and interquartile ranges for continuous variables with a skewed distribution (Townsend deprivation index, alcohol consumption, fibrinogen, ferritin, plasma glucose, cholesterol, low-density lipoprotein, high-density lipoprotein, triglycerides), and percentages for binary variables. Among participants with positive tests, a *P* value for linear trend in the baseline characteristic across thirds of the distribution was calculated using linear regression (continuous variables) or logistic regression (binary variables), adjusted for age and sex. Continuous variables with skewed distributions were log transformed. Similar methods were used to test the difference between participants with positive and negative tests.

We used Cox proportional hazards regression models to estimate age- and sex-adjusted mortality rates (using the age/sex distribution of our dataset as the standard population) and hazard ratios of death, comparing seropositive vs seronegative individuals, and also comparing thirds of RLU values, with seronegative individuals as the reference group. We also estimated the effect of each exposure on cardiovascular death, cancer-related death, and noncancer, noncardiovascular-related death, separately. The assumption of proportional hazards was tested by plotting and inspecting the relevant Kaplan-Meier survival curves.

Within the Cox model we tested for interactions between the exposure of interest (either seropositive vs seronegative or thirds of RLU values) and sex, age at time of entry to the study (continuous), socioeconomic status (manual vs nonmanual employment), physical activity, body mass index (BMI; continuous), and inflammatory markers (ferritin, fibrinogen, and CRP, all continuous). We fit multivariable models adjusting for various potential confounders. We did not assume any mediation effects.

Covariates were chosen as possible confounders (age, sex, Townsend deprivation index, smoking, educational level, physical activity, social class, BMI, waist-to-hip ratio [WHR], total cholesterol, CRP) based on a priori hypotheses only. We additionally performed all of the above analyses (1) including the 91 participants with the equivocal results initially in the seronegative and subsequently in the seropositive group (lower antibody category), and (2) excluding all seronegative individuals and repeating all the analyses within the seropositive group only, using the lower antibody group as the baseline. All statistical analyses were performed using Stata/SE 12.0 (StataCorp, College Station, Texas).

## RESULTS

A total of 59% of the participants were seropositive for CMV, with seropositivity being slightly more common in women (60%) compared to men (57%, χ^2^ *P* < .001) and at older ages. Higher CMV IgG antibody levels among seropositive participants were associated with older age and female sex (Table 1).

**Table 1.** Baseline Demographic, Lifestyle, and Comorbidity Characteristics of Participants of the European Prospective Investigation of Cancer–Norfolk Cohort Cytomegalovirus (CMV) Study (N = 12 999), Grouped by CMV Antibody Status

Characteristic	Negative for CMV Antibodies (n = 5366)	Positive for CMV IgG Antibodies				*P* Value^b^
		Low Antibody Group (n = 2545)	Middle Antibody Group (n = 2544)	High Antibody Group (n = 2544)	*P* Value^a^	
Age at study entry, y, mean (SD)	56.9 (9)	59.0 (9)	59.6 (9)	61.1 (9)	<.001	<.001
Female sex	53%	50%	56%	62%	<.001	<.001
Education, A level^c^ and above	58%	52%	52%	46%	.26	<.001
Social class, nonmanual employment	63%	58%	60%	58%	.97	<.001
Townsend deprivation index, median (IQR)	−2.7 (−3.7 to −1.3)	−2.6 (−3.6 to −1.0)	−2.6 (−3.7 to −1.0)	−2.5 (−3.7 to −0.9)	.61	<.001
Ever smokers	50%	56%	55%	56%	.11	<.001
Alcohol consumption, units/wk, median (IQR)	4.0 (1–10)	4.0 (1–10)	3.5 (1.9)	2.5 (1–8)	.08	.14
Physical activity, moderately or very active	44%	41%	41%	37%	.73	.25
With prevalent diabetes mellitus	2.5%	3.4%	3.3%	3.9%	.58	.04
With prevalent myocardial infarction	2.6%	3.4%	3.7%	4.2%	.10	.03
With prevalent stroke	1.2%	1.3%	1.6%	1.8%	.42	.87
With prevalent cancer, excluding nonmelanoma skin cancer	4.7%	4.6%	5.6%	5.8%	.35	.95

Abbreviations: CMV, cytomegalovirus; IgG, immunoglobulin G; IQR, interquartile range; SD, standard deviation.

^a^ Age- and sex-adjusted *P* value for trend among thirds of antibody within participants with positive IgG antibodies.

^b^ Age- and sex-adjusted *P* value comparing participants with positive IgG antibodies to participants with negative IgG antibodies.

^c^ “A levels” corresponds to 12 years of school education in the United Kingdom.

After age and sex adjustment, positive IgG antibody levels for CMV were associated with markers of lower social status, lifetime exposure to smoking, higher BMI and WHR, higher total cholesterol, and a history of diabetes mellitus and myocardial infarction (Tables 1 and 2). Among seropositive participants and after age and sex adjustment, there was an association between higher IgG antibody levels and higher total cholesterol, lower high-density lipoprotein, and higher triglycerides.

**Table 2.** Baseline Anthropometric and Biochemical Characteristics of Participants of the European Prospective Investigation of Cancer–Norfolk Cohort Cytomegalovirus (CMV) Study (N = 12 999), Grouped by CMV Antibody Status

	Negative for CMV Antibodies (n = 5366)	Positive for CMV IgG Antibodies				*P* Value^b^
		Low Antibody Group (n = 2545)	Middle Antibody Group (n = 2544)	High Antibody Group (n = 2544)	*P* Value^a^	
CMV IgG antibody, IU/mL, median (IQR)	0.2 (0.2–0.2)	3.0 (1.9–4.1)	6.9 (5.7–8.4)	14.1 (10.7–20.8)	<.001	<.001
Body mass index, kg/m^2^, mean (SD)	26.1 (3.7)	26.4 (3.7)	26.3 (3.9)	26.5 (4.1)	.45	<.001
Waist-to-hip ratio, mean (SD)	0.85 (0.1)	0.86 (0.1)	0.86 (0.1)	0.86 (0.1)	.29	.02
C-reactive protein, mg/L, mean (SD)	2.9 (5.9)	3.0 (5.6)	3.3 (6.5)	3.5 (7.6)	.05	.07
Ferritin, pmol/L, mean (SD)	206.5 (172)	206.4 (170)	204.6 (172)	194.1 (161)	.74	.14
Fibrinogen, µmol/L, median (IQR)	8.3 (7.1–9.8)	8.5 (7.1–9.9)	8.5 (7.1–9.9)	8.8 (7.4–9.9)	.18	.61
Glucose, mmol/L, median (IQR)	4.0 (3.5–4.7)	4.0 (3.5–4.7)	4.1 (3.5–4.8)	4.1 (3.6–4.8)	.26	.77
Cholesterol, mmol/L, median (IQR)	6.0 (5.3–6.8)	6.1 (5.4–6.9)	6.1 (5.4–6.9)	6.1 (5.4–6.9)	.07	.15
LDL, mmol/L, median (IQR)	3.8 (3.2–4.6)	3.9 (3.3–4.6)	3.8 (3.2–4.6)	3.9 (3.3–4.6)	.13	.63
HDL, mmol/L, median (IQR)	1.4 (1.1–1.7)	1.4 (1.1–1.6)	1.3 (1.1–1.6)	1.4 (1.1–1.7)	.001	<.001
Triglycerides, mmol/L, median (IQR)	1.5 (1.0–2.2)	1.5 (1.1–2.2)	1.6 (1.1–2.3)	1.6 (1.1–2.3)	.64	<.001
Systolic blood pressure, mm Hg, mean (SD)	134.2 (17)	135.1 (18)	135.9 (18)	137.2 (19)	.05	.59
Diastolic blood pressure, mm Hg, mean (SD)	82.1 (11)	82.3 (11)	82.5 (11)	82.9 (11)	.09	.89

To obtain cholesterol, LDL, and HDL in mg/dL, divide mmol/L by 0.0259. To obtain triglycerides in mg/dL, divide mmol/L by 0.0113. To obtain glucose in mg/dL, divide mmol/L by 0.0555. To obtain fibrinogen in mg/dL, divide µmol/L by 0.0294. To obtain ferritin in ng/mL, divide pmol/L by 2.247.

Abbreviations: CMV, cytomegalovirus; HDL, high-density lipoprotein; IgG, immunoglobulin G; IQR, interquartile range; LDL, low-density lipoprotein; SD, standard deviation.

^a^ Age- and sex-adjusted *P* value for trend among thirds of antibody within participants with positive IgG antibodies.

^b^ Age- and sex-adjusted *P* value comparing participants with positive IgG antibodies to participants with negative IgG antibodies.

### Mortality

After a mean of 14.3 years (SD, 3.3) of follow-up, 2514 deaths occurred. Age- and sex-adjusted mortality rates were 12.4 (95% confidence interval [CI], 11.3–13.2) per 1000 person-years at risk among seronegative participants and 14.2 (95% CI, 13.5–15.0) among seropositive participants. Among seropositive participants, rates increased across thirds of IgG antibody levels (score test of trend for rates *P* < .0001). A total of 851 deaths were attributed to cardiovascular diseases and 955 to cancer, and 708 had an underlying cause other than cancer or cardiovascular disease. Of the noncardiovascular, noncancer-related deaths, 12% (n = 76) had respiratory diseases, 16% (n = 100) had gastrointestinal diseases, and 21% (n = 130) had central and peripheral nervous system diseases coded as the underlying cause. The rest of the deaths were attributed to various causes including infection, kidney, blood, joint, endocrine, and rheumatologic diseases as well as alcohol abuse or intoxication and accidents.

### Associations Between CMV and All-Cause Mortality

CMV seropositivity was associated with all-cause mortality, after age and sex adjustment as well as after adjustment for socioeconomic factors and BMI, WHR, total cholesterol, and CRP (Table 3). Hazard ratios for mortality were higher for the group of participants with the highest antibody levels for all of the models examined (Table 3). Hazard ratios for mortality were similar after excluding participants who died within 2 years from study entry or had prevalent myocardial infarction, cerebrovascular accident, or cancer at study entry (Table 3). No interactions with sex, age, socioeconomic status, physical activity, BMI, and inflammatory markers were identified. A statistically significant association was noted between death not attributed to cancer and cardiovascular disease and CMV seropositivity (Table 4). Hazard ratios for mortality were identical to those in Tables 3 and 4 when the 91 participants with the equivocal results were included in the seronegative or in the seropositive lower antibody category group. Limiting the analyses within the seropositive participants only and using the low antibody group as the reference category confirmed that participants with higher antibody groups had higher hazard ratios for all-cause and cause-specific mortality (Supplementary Tables 1 and 2).

**Table 3.** Hazard Ratios for All-Cause Mortality

Model	Model Adjustments	No.	All With Positive CMV IgG Antibodies	Low Antibody Group	Middle Antibody Group	High Antibody Group
Model 1	Age, sex	12 999	1.16 (1.07–1.26)	1.09 (.98–1.22)	1.14 (1.02–1.27)	1.26 (1.13–1.39)
Model 2	Age, sex, Townsend, smoking, educational level, physical activity, social class	12 612	1.14 (1.04–1.24)	1.07 (.95–1.20)	1.12 (.99–1.25)	1.23 (1.10–1.37)
Model 3	Age, sex, Townsend, smoking, educational level, physical activity, social class, BMI, WHR, total cholesterol	12 425	1.15 (1.05–1.25)	1.08 (.96–1.21)	1.13 (1.01–1.27)	1.24 (1.11–1.38)
Model 4	Age, sex, Townsend, smoking, educational level, physical activity, social class, BMI, WHR, total cholesterol, CRP	11 840	1.13 (1.03–1.24)	1.06 (.94–1.19)	1.11 (.99–1.24)	1.23 (1.09–1.37)
Model 4 sensitivity analysis	Excluding deaths during first 2 y of follow-up	11 707	1.12 (1.02–1.23)	1.06 (.94–1.19)	1.08 (.96–1.22)	1.23 (1.09–1.38)
	Participants without baseline cancer, MI, or CVA	10 684	1.11 (1.00–1.23)	1.06 (.93–1.21)	1.08 (.95–1.23)	1.19 (1.06–1.36)

Data are presented as hazard ratio (95% confidence interval). Reference group is participants with negative IgG antibody for CMV, the European Prospective Investigation of Cancer–Norfolk cohort CMV study (N = 12 999). All models are adjusted for age at recruitment to the study, sex, Townsend deprivation index [32], smoking, educational level, physical activity, social class, body mass index, waist-to-hip ratio, total cholesterol, C-reactive protein (see table below).

Abbreviations: BMI, body mass index; CMV, cytomegalovirus; CRP, C-reactive protein; CVA, cerebrovascular accident; IgG, immunoglobulin G; MI, myocardial infarction; Townsend, Townsend deprivation index [32]; WHR, waist-to-hip ratio.

**Table 4.** Hazard Ratios for Mortality Grouped by Different Attributable Cause and Seropositivity and Immunoglobulin G Antibody Levels for Cytomegalovirus

Model Outcome	No. of Deaths	All With Positive CMV IgG Antibodies	Low Antibody Group	Middle Antibody Group	High Antibody Group
Death attributed to cardiovascular diseases	851	1.06 (.91–1.24)	1.02 (.83–1.25)	0.91 (.75–1.13)	1.24 (1.03–1.49)
Death attributed to cancer	955	1.13 (.98–1.31)	1.09 (.90–1.31)	1.18 (.99–1.42)	1.13 (.94–1.36)
Death attributed to causes other than cardiovascular diseases or cancer	708	1.23 (1.04–1.47)	1.09 (.87–1.37)	1.25 (1.01–1.55)	1.35 (1.09–1.67)

Data are presented as hazard ratio (95% confidence interval). Reference group is participants with negative IgG antibody, the European Prospective Investigation of Cancer–Norfolk cohort CMV study (N = 12 999). All models are adjusted for age at recruitment to the study, sex, Townsend deprivation index [32], smoking, educational level, physical activity, social class, body mass index, waist-to-hip ratio, total cholesterol, C-reactive protein.

Abbreviations: CMV, cytomegalovirus; IgG, immunoglobulin G.

## DISCUSSION

This is the largest ever population-based study examining all-cause and specific-cause mortality in association with CMV IgG antibody levels.

Our findings support an independent association between seropositivity for CMV and 14-year mortality among adult participants, as shown by the previous population study [16] as well as previous small studies limited to older women [9], patients with high-level stenosis of the coronary arteries and high CRP [10], or patients undergoing coronary angiography [11]. Our findings also support an association between the level of CMV IgG antibodies and mortality.

Higher CMV IgG antibody levels as well as CMV DNA in the urine but not in the blood are more frequently found among older individuals [24], suggesting asymptomatic CMV reactivation in older age. CMV-specific antibody levels correlate well with the numbers of circulating CMV-specific memory B cells and are not due to a generally higher antibody production by the individuals [25]. Previous studies have attempted to examine the association between CMV IgG antibody levels and mortality. These studies have been limited to highly selected groups including Latinos over the age of 60 [13], community-dwelling older women [14], and community-dwelling older adults with stable cardiovascular disease [15]. All 3 studies found evidence of an association between higher antibody levels and mortality but had a low discriminatory power to examine the effect of seropositivity vs seronegativity because of prevalent seropositivity, and 2of them grouped participants negative for CMV IgG antibody into the lower antibody level groups, making interpretations about any possible effect of CMV difficult. Our study shows that high CMV antibody levels are associated with all-cause mortality.

CMV seropositivity is thought to contribute to oncogenesis [26, 27], but any excess of cancer-related deaths is not apparent in a population study of this size. Seropositivity for CMV and CMV latency has been associated with telomere shortening [28], making it possible that CMV could be contributing to mortality through its effects on immunity. The relations that we observe appear to be independent of CRP level, suggesting that we are not observing a general association with other intercurrent infection or inflammation.

Measurement error might have affected our results. The assay used is not affected by other common viral antibodies and has good performance characteristics [29]. Error might have also been introduced by inaccuracies in death certificate information, although it is unlikely that such error would have been differential by CMV antibody status.

Residual confounding might have also affected our results. In this study we measured CMV IgG antibody titers at a single time point and did not directly measure CMV reactivation. Although it is postulated that higher CMV IgG antibody levels represent more frequent or intense subclinical CMV reactivation from latency, this has not been conclusively proven. CMV IgG antibody levels correlate well with CMV-specific B-cell numbers [25], but significant intraindividual variation exists [25], and antibody levels do not always reflect the presence of CMV DNA [30]. Other events that might affect CMV IgG levels acutely or chronically have not been studied. It is possible, therefore, that CMV IgG antibody levels are elevated through an unknown mechanism and that the underlying cause is also associated with the risk of death. In this context, CMV infection might even be an innocent bystander or a measure of a failing immune system [31]. The true cause of increased mortality is less likely to be contemporary social deprivation, as previously hypothesized, as after adjusting for multiple variables associated with social status in our analysis, the observed hazard ratios did not appreciably change.

## CONCLUSIONS

This is the first population cohort study to show an association between seropositivity as well as CMV antibody level and mortality. We show that the association with all-cause mortality is not explained by various measures of social deprivation or inflammation and is not limited to cardiovascular disease. Studies attempting to correlate the levels of IgG CMV antibody with longitudinal measurements of viral load or other direct measurements of viral reactivation will be necessary to further explore the strong observed associations.

## Supplementary Data

Supplementary materials are available at *Clinical Infectious Diseases* online (http://cid.oxfordjournals.org/). Supplementary materials consist of data provided by the author that are published to benefit the reader. The posted materials are not copyedited. The contents of all supplementary data are the sole responsibility of the authors. Questions or messages regarding errors should be addressed to the author.

Supplementary Data
