# Supplementary material for: Seropositivity and Higher Immunoglobulin G Antibody Levels Against Cytomegalovirus Are Associated With Mortality in the Population-Based European Prospective Investigation of Cancer–Norfolk Cohort
Source: Clin Infect Dis. 2013 Feb 26;56(10):1421–7. doi: 10.1093/cid/cit083 (PMC3634310; doi:10.1093/cid/cit083)
Supplement: Supplementary Data [file supp_56_10_1421__index.html]

Seropositivity and Higher Immunoglobulin G Antibody Levels Against Cytomegalovirus Are Associated With Mortality in the Population-Based European Prospective Investigation of Cancer–Norfolk Cohort — Seropositivity and Higher Immunoglobulin G Antibody Levels Against Cytomegalovirus Are Associated With Mortality in the Population-Based European Prospective Investigation of Cancer–Norfolk Cohort — Supplementary Data 

# Seropositivity and Higher Immunoglobulin G Antibody Levels Against Cytomegalovirus Are Associated With Mortality in the Population-Based European Prospective Investigation of Cancer–Norfolk Cohort

## Supplementary Data

Supplementary Data

**Files in this Data Supplement:**

- Supplementary Data - Doc file
