# Supplementary material for: Seropositivity and Higher Immunoglobulin G Antibody Levels Against Cytomegalovirus Are Associated With Mortality in the Population-Based European Prospective Investigation of Cancer–Norfolk Cohort
Source: Clin Infect Dis. 2013 Feb 26;56(10):1421–7. doi: 10.1093/cid/cit083 (PMC3634310; doi:10.1093/cid/cit083)
Supplement: Supplementary Data [file supp_cit083_cit083supp.doc]

| **Supplementary Table 1. Hazard Ratios for all-cause mortality limited within participants who are seropositive. Reference group is participants with low IgG antibody for CMV (“low antibody group”) , The EPIC-Norfolk cohort CMV Study (N= 7,633)** | | | | | |
| --- | --- | --- | --- | --- | --- |
|  | **Model adjustments** | N | Low antibody group | Middle antibody group | High antibody group |
| **Model 1** | Age, sex | 7,633 | 1 | 1.04 (0.92, 1.17) | 1.15 (1.03, 1.29) |
| **Model 2** | Age, sex, Townsend, Smoking, educational level, physical activity, social class | 7,404 | 1 | 1.05 (0.92, 1.18) | 1.16 (1.03, 1.31) |
| **Model 3** | Age, sex, Townsend, Smoking, educational level, physical activity, social class, BMI, WHR, total cholesterol | 7,294 | 1 | 1.05 (0.93, 1.19) | 1.16 (1.02, 1.31) |
| **Model 4** | Age, sex, Townsend, Smoking, educational level, physical activity, social class, BMI, WHR, total cholesterol, CRP | 6,955 | 1 | 1.05 (0.92, 1.19) | 1.17 (1.03, 1.32) |
| Age is age at recruitment to the study. Smoking: lifetime history of smoking. BMI: Body-Mass Index. WHR: Waist-to-Hip Ratio. CRP: C-Reactive Protein. CVA: Cerebrovascular Accident. MI: Myocardial Infarction. Townsend: Townsend Deprivation Index . | | | | | |

| **Supplementary Table 2. Hazard Ratios for mortality grouped by different attributable cause limited within participants who are seropositive. Reference group is participants with low IgG antibody for CMV (“low antibody group”) , The EPIC-Norfolk cohort CMV Study (N= 7,633)** | | | | |
| --- | --- | --- | --- | --- |
| **Model Outcome** | N of deaths | Low antibody group | Middle antibody group | High antibody group |
| **Death attributed to Cardiovascular diseases** | 569 | 1 | 0.90 (0.71, 1.13) | 1.21 (0.99, 1.50) |
| **Death attributed to Cancer** | 626 | 1 | 1.05 (0.88, 1.30) | 1.10 (0.89, 1.35) |
| **Death attributed to causes other than Cardiovascular Diseases or Cancer** | 492 | 1 | 1.16 (0.91, 1.48) | 1.25 (0.99, 1.59) |
| All models are adjusted for Age at recruitment to the study, Sex, Townsend Deprivation Index , Smoking, Educational level, Physical Activity, Social Class, Body-Mass Index, Waist-to-Hip ratio, total Cholesterol, C-Reactive Protein. | | | | |
